# Clinical Role of ASCT2 (SLC1A5) in KRAS-Mutated Colorectal Cancer

**DOI:** 10.3390/ijms18081632

**Published:** 2017-07-27

**Authors:** Kosuke Toda, Gen Nishikawa, Masayoshi Iwamoto, Yoshiro Itatani, Ryo Takahashi, Yoshiharu Sakai, Kenji Kawada

**Affiliations:** Department of Surgery, Graduate School of Medicine, Kyoto University, Kyoto 606-8507, Japan; kotoda@kuhp.kyoto-u.ac.jp (K.T.); gnishika@kuhp.kyoto-u.ac.jp (G.N.); iwamoto@kuhp.kyoto-u.ac.jp (M.I.); itatani@kuhp.kyoto-u.ac.jp (Y.I.); ryotak@kuhp.kyoto-u.ac.jp (R.T.); ysakai@kuhp.kyoto-u.ac.jp (Y.S.)

**Keywords:** colorectal cancer, KRAS, ASCT2, SLC1A5

## Abstract

Mutation in the *KRAS* gene induces prominent metabolic changes. We have recently reported that *KRAS* mutations in colorectal cancer (CRC) cause alterations in amino acid metabolism. However, it remains to be investigated which amino acid transporter can be regulated by mutated *KRAS* in CRC. Here, we performed a screening of amino acid transporters using quantitative reverse-transcription polymerase chain reaction (RT-PCR) and then identified that ASCT2 (*SLC1A5*) was up-regulated through KRAS signaling. Next, immunohistochemical analysis of 93 primary CRC specimens revealed that there was a significant correlation between *KRAS* mutational status and ASCT2 expression. In addition, the expression level of ASCT2 was significantly associated with tumor depth and vascular invasion in *KRAS*-mutant CRC. Notably, significant growth suppression and elevated apoptosis were observed in *KRAS*-mutant CRC cells upon *SLC1A5*-knockdown. ASCT2 is generally known to be a glutamine transporter. Interestingly, *SLC1A5*-knockdown exhibited a more suppressive effect on cell growth than glutamine depletion. Furthermore, *SLC1A5*-knockdown also resulted in the suppression of cell migration. These results indicated that ASCT2 (*SLC1A5*) could be a novel therapeutic target against *KRAS*-mutant CRC.

## 1. Introduction

Colorectal cancer (CRC) is one of the most common cancers worldwide; therefore, development of novel diagnostic measures and treatment is very important [1]. *KRAS* mutations are found in approximately 40% of CRC cases [2,3,4]. A number of clinical trials have shown that *KRAS* mutations in CRC can predict a lack of responses towards anti-epidermal growth factor receptor (EGFR)-based therapy [2,3,4]. Therefore, development of new therapy for CRC with mutated *KRAS* has been desired clinically. Some studies have investigated the correlation between *KRAS* mutations and metabolic alterations in pancreatic and lung cancers [5,6,7,8,9] as well as in CRC [10,11,12,13,14,15]. We have recently reported that, using metabolome analysis, concentration of amino acids is elevated in CRC cells with mutated *KRAS* compared to CRC cells with wild-type *KRAS* [12]. The increase in glucose transporter 1 (GLUT1) expression and glucose uptake was critically dependent on mutated *KRAS* [16,17,18]. However, it remains to be investigated which amino acid transporter is specifically regulated by mutated *KRAS* in CRC. In the present study, we performed a screening of amino acid transporters in *KRAS*-mutant CRC cells transfected by si*KRAS* and found that ASCT2 (*SLC1A5)* was particularly up-regulated through KRAS signaling.

The *SLC1A5* gene encodes alanine-serine-cysteine amino acid transporter (ASCT2), which is an essential glutamine transporter. ASCT2 over-expression has been reported in several cancers [19,20,21,22,23,24,25,26,27,28,29,30,31]. However, the role of ASCT2 in CRC has not yet been reported. In addition to glucose, glutamine is an essential source of cellular building blocks to fuel cell proliferation. Recent studies have established a better understanding about the importance of glutamine as a critical nutrient in fast growing cancer cells [32,33,34]. In the present study, we investigated the significance of ASCT2 expression in CRC using in vitro cultures and clinical samples.

## 2. Results

### 2.1. SLC1A5 (ASCT2) Is Regulated through KRAS Signaling in KRAS-Mutant CRC Cells

**Figure 1:**
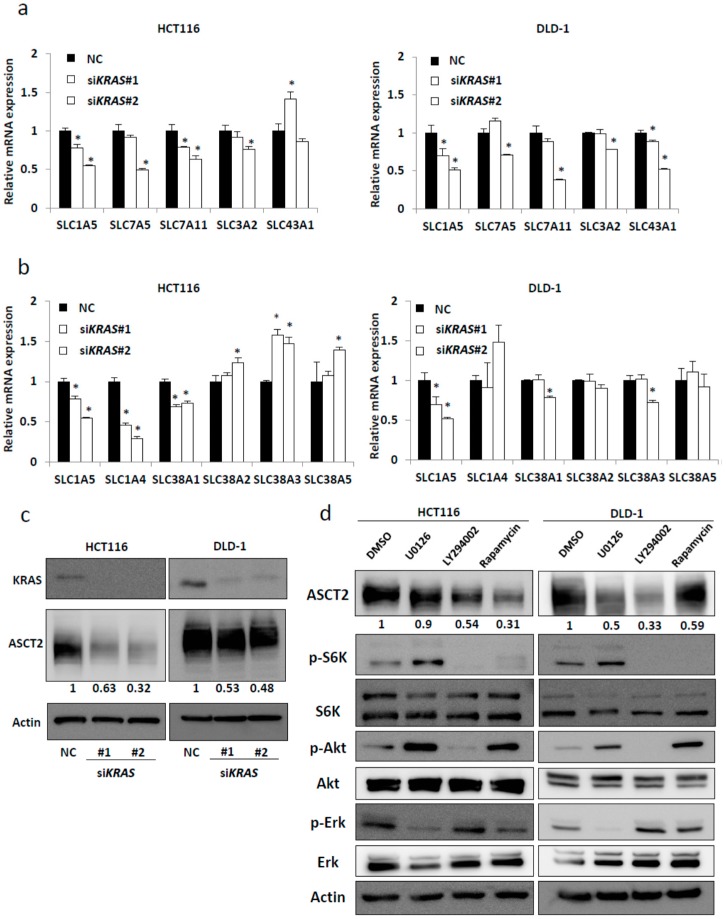
Identification of the amino acid transporter regulated by mutated *KRAS*. (**a**) Relative mRNA levels of amino acid transporters that are reported to be associated with cancer; (**b**) relative mRNA levels of amino acid transporters that are involved in glutamine transport. HCT116 cells (left) and DLD-1 cells (right) were treated separately with two independent siRNA constructs (#1 and #2) targeting *KRAS* and negative control (NC) siRNA. Mean; bars, ± SD, *n* = 3 (Student’s *t*-test; * *p* < 0.05); (**c**) Western blotting for KRAS, ASCT2, and β-actin (Actin). The relative ASCT2 expression levels for three independent experiments are shown by quantitative analysis normalized to β-actin (Actin); (**d**) CRC cells (HCT116 and DLD-1) were treated with 0.1% dimethyl sulfoxide (DMSO), 20 μM U0126 (MEK inhibitor), and 50 μM LY294002 (PI3K inhibitor) or 20 nM rapamycin (mTOR inhibitor) for 48 h. Protein levels of ASCT2 were normalized to β-actin (Actin). Densitometry values were expressed as fold change compared with DMSO-treated cells.

We have recently reported that mutated *KRAS* induces metabolic alterations in many amino acids [12]. Therefore, we hypothesized that the expression of amino acid transporters might be regulated by mutated *KRAS*. Several amino acid transporters (*SLC1A5*, *SLC7A5*, *SLC7A11*, *SLC3A2*, and *SLC43A1*) have been reported to be up-regulated in different cancers [32]. To determine the specific transporter that could be regulated by mutated *KRAS*, we introduced two different small interfering RNAs (siRNAs) targeting *KRAS* in *KRAS*-mutant CRC cell lines (HCT116 and DLD-1). We confirmed that si*KRAS* significantly reduced the mRNA levels of *KRAS* in both cell lines (Figure S1a). Interestingly, *KRAS*-knockdown significantly reduced *SLC1A5* expression in both *KRAS*-mutant cell lines (Figure 1a). *SLC1A5* (ASCT2) is a known glutamine transporter. Next, we investigated whether glutamine transporters other than *SLC1A5* (i.e., *SLC1A4*, *SLC38A1*, *SLC38A2*, *SLC38A3*, and *SLC38A5*) could be regulated by KRAS signaling [33]. Expression levels of *SLC1A4* and *SLC38A1* were decreased after *KRAS*-knockdown in HCT116; however, their expression was not decreased in DLD-1 (Figure 1b). We also found that *KRAS*-knockdown significantly reduced protein expression of ASCT2 in both *KRAS*-mutant cell lines (Figure 1c). The mutated *KRAS* continuously activates both Raf/MEK/ERK and PI3K/Akt/mTOR pathways. To investigate which pathway regulates ASCT2 expression, we used specific inhibitors of each pathway. Western blot analysis revealed that ASCT2 expression was dramatically reduced in *KRAS*-mutant CRC cell lines by addition of LY 294002 (PI3K inhibitor) or rapamycin (mTOR inhibitor), which suggested that KRAS signaling may regulate ASCT2 expression in CRC mainly via the PI3–Akt–mTOR pathway (Figure 1d).

### 2.2. Relationship between ASCT2 Expression and KRAS Mutational Status in CRC Clinical Samples

**Figure 2:**
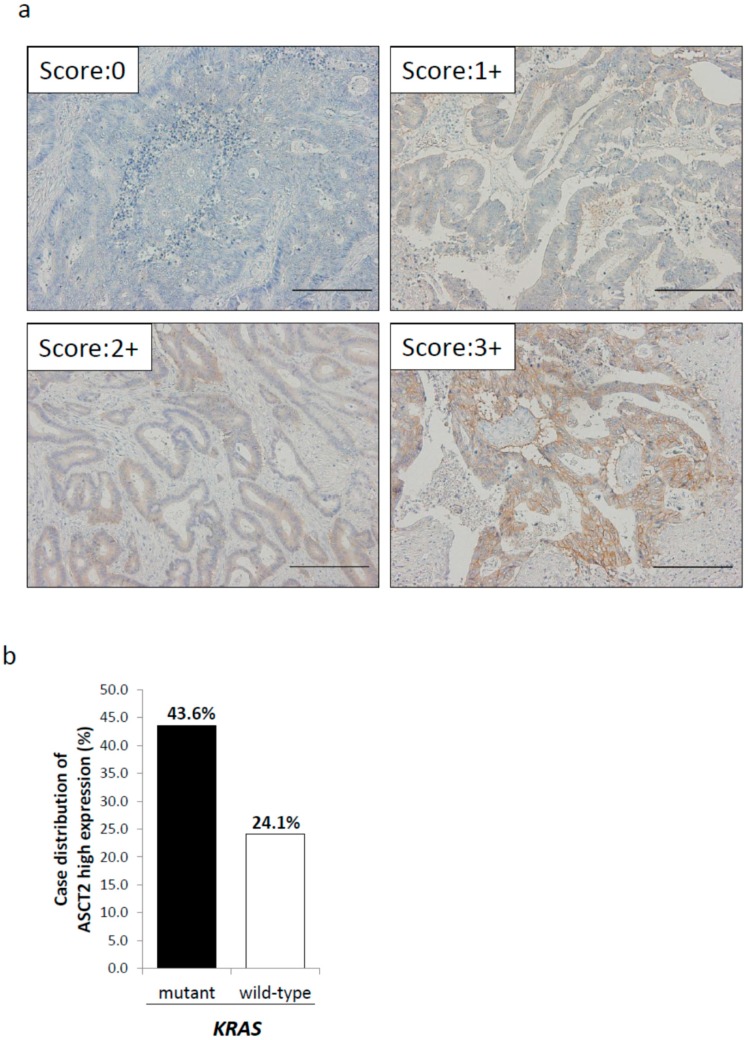
Immunohistochemical staining for ASCT2 of primary colorectal cancer (CRC) specimens. (**a**) Representative picture. Scale bar, 200 μm (200× magnification); (**b**) Relationship between *KRAS* mutational status and ASCT2 expression.

We next performed immunohistochemistry (IHC) to evaluate the relationship between ASCT2 expression and *KRAS* mutational status in clinical specimens of human primary CRC. Regarding the expression levels of ASCT2, we classified the clinical specimens into four groups; score 0 (0–10%), score 1+ (10–40%), score 2+ (40–70%), and score 3+ (≥70%). Score 0 was found in 12 patients (12.9%), score 1+ in 22 patients (23.6%), score 2+ in 29 patients (31.2%), and score 3+ in 30 patients (32.3%) (Figure 2a). We defined score 3+ as the high expression group, while score 0, 1+, and 2+ were categorized as the low expression group. Regarding *KRAS* mutational status, mutated *KRAS* and wild-type *KRAS* were found in 39 and 54 patients, respectively. ASCT2 expression was high in 43.6% (17 of 39) of CRC patients with mutated *KRAS*, whereas in 24.1% (13 of 54) of CRC patients with wild-type *KRAS*, which indicated that there was a significant correlation between high ASCT2 expression and *KRAS* mutation (risk ratio: 1.62, 95%; confidence interval (CI): 1.02–2.57, *p* = 0.047, Figure 2b).

### 2.3. Knockdown of SLC1A5 (ASCT2) Results in Suppression of Cell Growth

**Figure 3:**
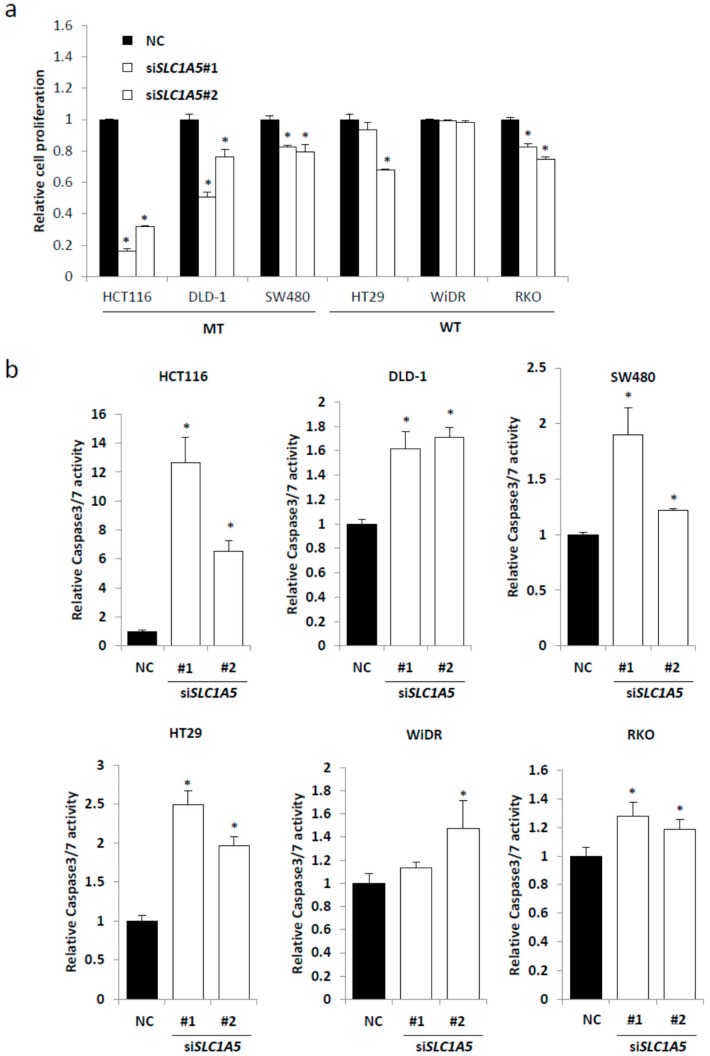
*SLC1A5* knockdown inhibits cell proliferation and induces cell apoptosis of CRC cells. (**a**) Cell proliferation measured by CCK-8 assay. CRC cells were transfected with negative control (NC) or two independent si*SLC1A5* and cultured for 72 h. Viability in each si*SLC1A5* was normalized to that in NC. Student’s *t*-test; * *p* < 0.05; (**b**) caspase 3/7 activities measured by Caspase-Glo assay. CRC cells transfected with negative control (NC) or two independent si*SLC1A5* were cultured for 72 h. Caspase 3/7 activity was normalized to the cell viability measured by CCK-8 assay under the same density and conditions. Mean; bars, ± SD, *n* = 3 (Student’s *t*-test; * *p* < 0.05).

To investigate the role of *SLC1A5* (ASCT2) in CRC cell lines with mutated *KRAS*, we introduced non-silencing siRNA and two different siRNAs targeting *SLC1A5* (referred as si*SLC1A5*#1 and si*SLC1A5*#2) into CRC cell lines (Figure S2). Knockdown of *SLC1A5* (ASCT2) significantly suppressed the cell growth in all the 3 cell lines with mutated *KRAS* (HCT116, DLD-1, and SW480), whereas in 1 out of 3 cell lines with wild-type *KRAS* (RKO) (Figure 3a). Furthermore, we investigated the knockdown effect of *SLC1A5* on cell apoptosis. Knockdown of *SLC1A5* induced a significant increase in caspase 3/7 activities in all the 3 cell lines with mutated *KRAS* (HCT116, DLD-1, and SW480), whereas in 2 out of 3 cell lines with wild-type *KRAS* (HT29 and RKO) (Figure 3b). Oncogenic *PIK3CA* mutations were reported to reprogram glutamine metabolism in CRC [35]. *PIK3CA* mutations are observed in HCT116 (a H1047R mutation), DLD-1 (E545K; D549N mutations), HT29 (a P449T mutation), WiDR (a P449T mutation), and RKO (a H1047R mutation), whereas the *PIK3CA* status is wild-type in SW480. The *PIK3CA* status might be related to the differences in the knockdown effect of *SLC1A5* between cell lines.

### 2.4. Role of SLC1A5 (ASCT2) in KRAS-Mutant CRC Cells

**Figure 4:**
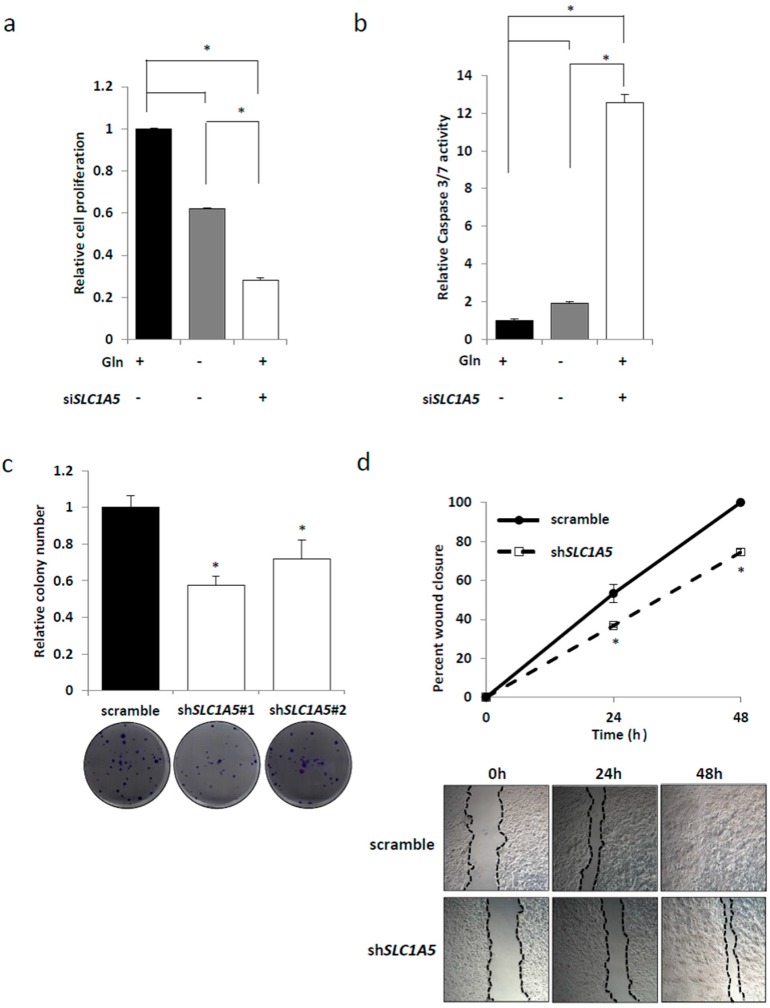
The role of *SLC1A5* (ASCT2) in *KRAS*-mutant CRC cells. *SLC1A5* knockdown exhibited more effective on suppressing cell growth (**a**) and inducing apoptosis (**b**) than under glutamine deprivation. Mean; bars, ± SD, *n* = 3 (Student’s *t*-test; * *p* < 0.05); (**c**) clonogenic assay with HCT116 transfected with control or two independent sh*SLC1A5* vectors. Cells were maintained under 4 mM glutamine condition containing 10% fetal bovine serum (FBS) for 10 days. Mean; bars, ± SD, *n* = 3 (Student’s *t*-test; * *p* < 0.05); (**d**) wound healing assay with HCT116 transfected with control or sh*SLC1A5* vector. Cells were photographed at 50× magnification at 0, 24, and 48 h. Wound closure (%) was evaluated. Mean; bars, ± SD, *n* = 3 (Student’s *t*-test; * *p* < 0.05).

*SLC1A5* (ASCT2) is generally regarded as a glutamine transporter. In a *KRAS*-mutant CRC cell line (HCT116), glutamine depletion resulted in decreased cell proliferation and enhanced caspase 3/7 activities. Importantly, even in the presence of glutamine, si*SLC1A5* dramatically suppressed cell proliferation and up-regulated caspase 3/7 activities (Figure 4a,b), which indicated that the effect of *SLC1A5*-knockdown was more prominent on cell growth and apoptosis than glutamine depletion. To further investigate the functional role of *SLC1A5*, we established stable HCT116 transfectant cell lines in which *SLC1A5* was knocked down by shRNA constructs targeting *SLC1A5* (referred as sh*SLC1A5*#1 and sh*SLC1A5*#2) (Figure S3). In the clonogenic assay, *SLC1A5*-knockdown significantly suppressed colony number as compared to the control (Figure 4c). Moreover, in the wound healing assay, *SLC1A5*-knockdown significantly inhibited wound closure as compared to the control (Figure 4d). Taken together, these results indicate that the inhibition of *SLC1A5* (ASCT2) could be a therapeutic target in *KRAS*-mutant CRC.

### 2.5. Tumor Characteristics and ASCT2 Expression in CRC Clinical Samples

Table 1 shows the relationship between ASCT2 expression and clinicopathologic variables. ASCT2 expression was significantly correlated with tumor location, but not with age, sex, tumor size, stage, T-/N-/M-category, lymphatic invasion, or vascular invasion. We further investigated the clinical significance of ASCT2, based on the *KRAS* mutational status. Interestingly, we found that high ASCT2 expression was significantly associated with tumor depth and vascular invasion in *KRAS*-mutant CRC, which was not observed in wild-type *KRAS* CRC.

### 2.6. Patients’ Prognosis

**Figure 5:**
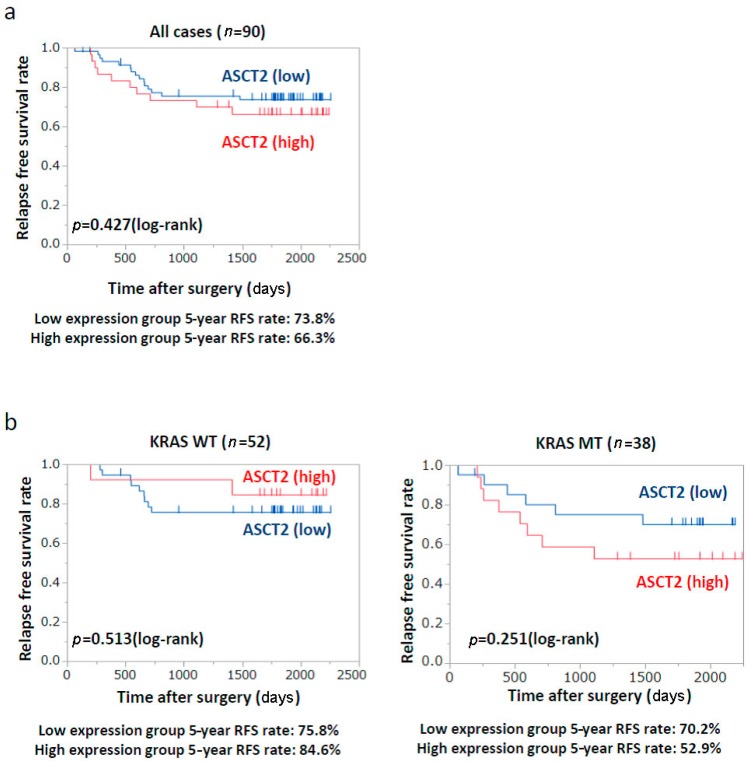
Kaplan–Meier analysis of relapse-free survival (RFS) according to ASCT2 expression and *KRAS* status. (**a**) RFS according to ASCT2 expression in total patients; (**b**) RFS according to ASCT2 expression in *KRAS*-mutant cases (right) and wild-type *KRAS* cases (left).

To evaluate the relationship between ASCT2 expression and patients’ prognosis, we performed the log-rank test analysis with CRC patients who underwent curative resection of primary CRC (*n* = 90). Kaplan–Meier survival curves indicated that ASCT2 expression was not significantly correlated with recurrence-free survival (RFS) in all cases (Figure 5a). However, in *KRAS*-mutant CRC cases (*n* = 38), the estimated RFS rate at 5-year tended to be lower in the high ASCT2 group than in the low ASCT2 group (52.9% vs. 70.2%; *p* = 0.251) (Figure 5b, right). On the other hand, in wild-type *KRAS* CRC cases (*n* = 52), the estimated RFS rate at 5-year was almost similar between the high and low ASCT2 groups (84.6% vs. 75.8%; *p* = 0.513) (Figure 5b, left). Taken together, high ASCT2 expression can be one of the crucial prognostic factors in *KRAS*-mutant CRC.

## 3. Discussion

*KRAS* mutations are found in a variety of human cancers, including pancreatic cancer, non-small cell lung cancer, and CRC. Recent studies have shown that mutated *KRAS* promotes metabolic reprogramming through nutrients uptake, glycolysis, glutaminolysis, and synthesis of nucleotides and fatty acids. The mechanism by which mutated *KRAS* coordinates the metabolic reprogramming to promote tumor growth remains to be investigated. The International CRC Subtyping Consortium has suggested that CRC can be divided into four subtypes with distinguished features: CMS1, CMS2, CMS3, and CMS4 [15]. Notably, CMS3 is characterized by metabolic dysregulation and is strongly associated with *KRAS* mutations. Using a comprehensive metabolomics analysis with isogenic CRC cell lines harboring mutated or wild-type *KRAS*, we have recently reported that mutated *KRAS* induces some metabolic alterations in glycolysis, the pentose phosphate pathway (PPP), the tricarboxylic acid (TCA) cycle, and most significantly in the amino acid pathway [12]. We identified that mutated *KRAS* regulated asparagine synthetase (ASNS), an enzyme that is involved in de novo synthesis of asparagine from aspartate, and that *KRAS*-mutant CRC cells could become adaptive to glutamine depletion through ASNS-dependent asparagine biosynthesis. There is also some evidence from other groups that *KRAS* mutations in CRC are associated with glutamine metabolism. Wong et al. reported that *SLC25A22* (a mitochondrial glutamine transporter) was a synthetic lethal metabolic gene in *KRAS*-mutant CRC cells and that expression of *SLC25A22* was correlated with poor prognosis in patients harboring *KRAS* mutations [11]. Miyo et al. reported that glutamine dehydrogenase 1 (GLUD1) and *SLC25A13* (a mitochondrial aspartate-glutamate carrier) played an essential role in cell survival of CRC cells under glucose-deprived conditions, and that combined expression of GLUD1 and *SLC25A13* was significantly associated with tumor aggressiveness and poorer prognosis in CRC patients [13]. These results indicate that the amino acid metabolism including glutaminolysis is more essential for cell survival in *KRAS*-mutant CRC than in wild-type *KRAS* CRC.

In this study, we focused on the amino acid transporter which was exclusively regulated by mutated *KRAS*, although several amino acid transporters have been reported to be up-regulated in cancer [32]. Herein, we identified *SLC1A5* as a novel target gene regulated by mutated *KRAS* in CRC. Expressions of *SLC25A22* and *SLC25A13* were not affected by *KRAS-*knockdown in our experiments (Figure S1b). Up-regulation of *SLC1A5* (ASCT2) and its clinical significance has been reported in a variety of human cancers [19,20,21,22,23,24,25,26,27,28,29,30,31]. In the present study, we demonstrated that *SLC1A5* (ASCT2) expression was regulated through KRAS signaling, and that *SLC1A5*-knockdown resulted in reduced cell growth and increased cell apoptosis in *KRAS*-mutant CRC cells (Figure 1 and Figure 3). Importantly, the effect of *SLC1A5*-knockdown was more prominent on cell growth and apoptosis than that of glutamine depletion (Figure 4a,b), which indicates that *SLC1A5* (ASCT2) plays a critical role in the malignant progression of *KRAS*-mutant CRC. Furthermore, *SLC1A5*-knockdown resulted in the suppression of cell migration (Figure 4d). In primary CRC clinical specimens, we found that ASCT2 expression was significantly associated with tumor depth and vascular invasion in *KRAS*-mutant CRC, but not in wild-type *KRAS* CRC (Table 1). In conclusion, our data indicates that *SLC1A5* (ASCT2) could be a novel biomarker as well as a potential therapeutic target in *KRAS*-mutant CRC.

## 4. Materials and Methods

### 4.1. Cell Lines and Reagents

HCT116, DLD-1, SW480, SW620, HT29, RKO, and WiDR cells were obtained from American Type Culture Collection. All cell lines were cultured in Dulbecco’s Modified Eagle Medium (DMEM) (glucose 25 mM, glutamine 4 mM) (043-30085, Wako, Tokyo, Japan) supplemented with 10% FBS and penicillin–streptomycin. Media without glutamine were prepared by using glutamine-free DMEM (glucose 25 mM, glutamine 0 mM) (045-32245, Wako) supplemented with 10% FBS. The identity of each cell line was confirmed by STR analysis (Takara Bio, Shiga, Japan). U0126 was purchased from Calbiochem, LY294002 and rapamycin were from Wako.

### 4.2. Quantitative Reverse Transcription Polymerase Chain Reaction (RT-PCR) Analysis

Total RNAs were extracted from cells with High Pure RNA Isolation Kit (Roche, Mannheim, Germany) according to the manufacturer’s instructions. RNA was reverse transcribed to cDNA with Transcriptor First Strand cDNA Synthesis Kit (Roche) according to the manufacturer’s instructions. The relative levels of respective genes were quantified using StepOnePlusTM Real-Time PCR System (Applied Biosystems, Foster City, CA, USA). The respective mRNA levels were normalized to that for ACTB. Primer sequences were found in Table S1.

### 4.3. Western Blot Analysis

Cells were washed with ice-cold phosphate-buffered saline and lysed in sodium dodecyl sulfate lysis buffer supplemented with inhibitor cocktails of protease and phosphatase. Primary antibodies can be found in Table S2.

### 4.4. Small Interfering RNA and Short Hairpin RNA

FlexiTube GeneSolutions for si*SLC1A5* (#1: SI05141017, #2: SI00079730) and non-silencing control siRNA (AllStars negative control siRNA, SI03650318) were purchased from Qiagen (Hilden, Germany). The siRNA (10 nM) was transfected with Lipofectamine RNAiMAX (Invitrogen, Carlsbad, CA, USA) according to the manufacturer’s reverse-transfection protocol. *SLC1A5* shRNA vectors were made from the same sequence of si*SLC1A5* (#1, #2), and cloned into pLKO.1 vectors. pLKO.1-scramble vector (Addgene) was used as control.

### 4.5. Cell Proliferation Assay

A cell proliferation assay was measured by Cell Counting Kit-8 (Dojindo, Kumamoto, Japan) according to the manufacturer’s instruction. Cells transfected with siRNA were cultured in 96-well plates at a density of 5000 cells/well for 72 h.

### 4.6. Clonogenic Assay

Cells were seeded in 6-well plates at a density of 100 cells per well in complete media. At the end point, colonies were fixed in 1% glutaraldehyde and stained with 0.2% crystal violet for 30 min, and number of colonies was counted. A colony was defined as a cluster of at least 50 cells.

### 4.7. Apoptosis Assay

The activity of Caspase-3 and -7 was measured by using Caspase-Glo 3/7 assay (Promega, Madison, WI, USA) according to the manufacturer’s protocol. Caspase activity was normalized to the cell number counted by CCK-8 cell proliferation assay under the same density and conditions.

### 4.8. Wound Healing Assay

Cell lines were seeded into 12-well plates and grew until 80–90% confluence. Confluent cultures were scratched with sterile tips, washed with PBS, and cultured in DMEM containing 5% FBS. Cells were photographed by a 50× magnification at 0, 24, and 48 h. Wound closure (%) was evaluated using the ImageJ software.

### 4.9. Immunohistochemistry

Formalin-fixed, paraffin-embedded sections were stained with anti-rabbit ASCT2 (Sigma-Aldrich, St. Louis, MO, USA) antibody. Antigen retrieval was achieved with microwave in citrate buffer (pH: 6.0). For primary CRC tissue, ASCT2 immunoreactivity score was determined by the proportion, as previously described [30]. The proportion was scored based on the positively rate as “0” (0–10%), “1” (10–40%), “2” (40–70%), “3” (>70%). Scores of 0, 1, and 2 were defined as low expression, whereas 3 was high expression.

Two researchers (Kosuke Toda and Gen Nishikawa) independently evaluated all immunohistochemistry samples without prior knowledge of other data. The slides with different evaluations among them were reinterpreted at a conference to reach the consensus.

### 4.10. Patients, Clinicopathological Data

93 patients were collected from patients who underwent primary colorectal cancer resection at Kyoto University Hospital between April 2009 and September 2013.

No patients received chemotherapy and/or radiation therapy. *KRAS* mutational status in all patients was analyzed by using an ABI 3130 Genetic Analyzer (Applied Biosystems, foster City, CA, USA), as described previously. Pathologic staging was categorized in accordance with the 7th edition of Union for International Cancer Control (UICC) classification of malignant tumors.

### 4.11. Statistical Analysis

All values were expressed as mean ± standard deviation (SD). Statistical analyses were conducted with the JMP Pro 12 (SAS Institute, Inc., Cary, NC, USA). Student’s *t*-test was used for comparing means between two groups. In clinical data, the statistical significance of differences between variables of two groups was determined by student’s *t*-test, chi-squared test, or Fisher’s exact test. Relapse-free survival (RFS) rates were evaluated by the Kaplan–Meier survival curve and log-rank test. All analyses were two-sided, and differences with a *p* value of less than 0.05 were considered statistically significant in all analyses.

## Figures and Tables

**Table 1:** Correlation between ASCT2 expression and clinicopathological variables (* *p* < 0.05).

Variables	Total			*KRAS* Wild-Type			*KRAS* Mutant		
	ASCT2			ASCT2			ASCT2		
	High (*n* = 30)	Low (*n* = 63)	*p-*value	High (*n* = 13)	Low (*n* = 41)	*p-*value	High (*n* = 17)	Low (*n* = 22)	*p-*value
Age, mean ± SD (*y*)	71.2 ± 9.4	68.0 ± 10.7	0.16	69.8 ± 10.5	67.2 ± 10.6	0.42	72.2 ± 9.8	69.4 ± 11.1	0.41
Sex									
Male	16	39	0.43	8	28	0.65	8	11	0.86
Female	14	24		5	13		9	11	
Location									
Left	18	50	0.049 *	9	33	0.45	9	17	0.11
Right	12	13		4	8		8	5	
Tumor size (mm)									
≥50	12	25	0.98	5	15	0.9	12	25	0.98
<50	18	38		8	26		18	38	
UICC-TMN stage									
I/II	14	33	0.61	7	23	0.89	7	10	0.79
III/IV	16	30		6	18		10	12	
T-category									
1/2	5	25	0.16	4	10	0.72	1	9	0.024 *
3/4	25	44		9	31		16	13	
M-category									
Negative	25	53	0.92	10	35	0.67	15	18	0.75
Positive	5	10		3	6		2	4	
N-category									
Negative	16	35	0.84	8	23	0.73	8	12	0.64
Positive	14	28		5	18		9	10	
Lymphatic invasion									
Negative	18	39	0.86	6	25	0.35	12	14	0.65
Positive	12	24		7	16		5	8	
Vascular invasion									
Negative	6	19	0.3	3	8	1	3	11	0.049 *
Positive	24	44		10	33		14	11	

## Supplementary Materials

Supplementary materials can be found at www.mdpi.com/1422-0067/18/8/1632/s1.

## Abbreviations

ASCT2	Alanine-serine-cysteine amino acid transporter
ASNS	Asparagine synthetase
CRC	Colorectal cancer
EGFR	Epidermal growth factor receptor
GLUD1	Glutamine dehydrogenase 1
GLUT1	Glucose transporter 1
PPP	Pentose phosphate pathway
RFS	Recurrence-free survival (RFS)
RT-PCR	Reverse-transcription polymerase chain reaction
siRNA	Small interfering RNA
TCA cycle	Tricarboxylic acid cycle
