# Supplementary material for: Clinical Role of ASCT2 (SLC1A5) in KRAS-Mutated Colorectal Cancer
Source: Int J Mol Sci. 2017 Jul 27;18(8):1632. doi: 10.3390/ijms18081632 (PMC5578022; doi:10.3390/ijms18081632)
Supplement: Supplementary file 1 [file ijms-18-01632-s001.zip › ijms-210244-supplementary table.pdf]

Table S1: Primer sequence for qRT-PCT

Gene name		Primer sequence
ACTB	forward	GCAAAGACCTGTACGCCAAC
ACTB	reverse	ACATCTGCTGGAAGGTGGAC
KRAS	forward	AGGCCTGCTGAAAATGACTG
KRAS	reverse	TGACCTGCTGTGTCGAGAAT
SLC1A5	forward	AAGCACATCAGCCGTTTCAT
SLC1A5	reverse	AGTGAGGACACCTCCAGCAG
SLC1A4	forward	ACGCAGGACAGATTTTCACC
SLC1A4	reverse	GAGGCAGGTCATGAGTAGGC
SLC7A5	forward	GTGTGTGATGACGCTGCTCT
SLC7A5	reverse	GCCAGGATGAAGAACACAGG
SLC7A11	forward	ACCATTCCCCTTGCAATATG
SLC7A11	reverse	CCAGTAGCCGCTCAGAAAAG
SLC3A2	forward	TCCAGGTTCGGGACATAGAG
SLC3A2	reverse	TGGATTCGAGTAGGCTCAGG
SLC43A1	forward	ACATGGCTGCTGTGAACAAG
SLC43A1	reverse	AGGTGAGAAGGCACAACAGC
SLC38A1	forward	CTGGCAAACACTGGAATCCT
SLC38A1	reverse	TCCCCCAGCTTTTCATACAC
SLC38A2	forward	CCACCTTAACACAGCCAACA
SLC38A2	reverse	ATGGGAAGAACAGCAGGATG
SLC38A3	forward	CACACCTACAGCAAGGTGGA
SLC38A3	reverse	GCTGAACTCCTGGTTTGGAA
SLC38A5	forward	GCTCACAGCAACCTTTGGAT
SLC38A5	reverse	ATAGGGAACAGCACGACTGG
SLC25A22	forward	CCAACGACTTCTTCCGACAT
SLC25A22	reverse	GCTGGATCTTCAGCATCTCC
SLC25A13	forward	AAAGCAAGCCTTTGTGCAAC
SLC25A13	reverse	ATGGGATGTGGTACCTCCAG

Table S2: Antibodies

Antibodies	Company	Phosphorylation site
mouse monoclonal anti-KRAS	Santa Cruz	
rabbit polyclonal anti-ASCT2	Santa Cruz	
rabbit monoclonal anti-pohospho-p44/42 kinase	Cell Signaling	Thr202/Tyr204
rabbit monoclonal anti-p44/42 kinase	Cell Signaling	
rabbit monoclonal anti-phospho-Akt	Cell Signaling	Ser473
rabbit monoclonal anti-Akt	Cell Signaling	
rabbit polyclonal anti-phospho-p70 S6 kinase	Cell Signaling	Thr389
rabbit polyclonal anti-p70 S6 kinase	Cell Signaling	
mouse monoclonal anti-β-actin-peroxidase	Sigma-Aldrich	
