# Supplementary material for: Clinical Role of ASCT2 (SLC1A5) in KRAS-Mutated Colorectal Cancer
Source: Int J Mol Sci. 2017 Jul 27;18(8):1632. doi: 10.3390/ijms18081632 (PMC5578022; doi:10.3390/ijms18081632)

Figure S1. The analysis of KRAS knockdown in CRC cell lines. (**a**) Quantitative RT-PCR showing relative mRNA levels for KRAS. HCT116 cells (left) and DLD-1 cells (right) were treated separately with two independent siRNA contructs (#1 and #2) targeting KRAS and negative control (NC) siRNA. Mean; bars, ± SD, n = 3 (Student's t-test; *p < 0.05); (**b**) Relative mRNA levels of SLC25A22 and SLC25A13. Mean; bars, ± SD, n = 3 (Student's t-test; *p < 0.05).

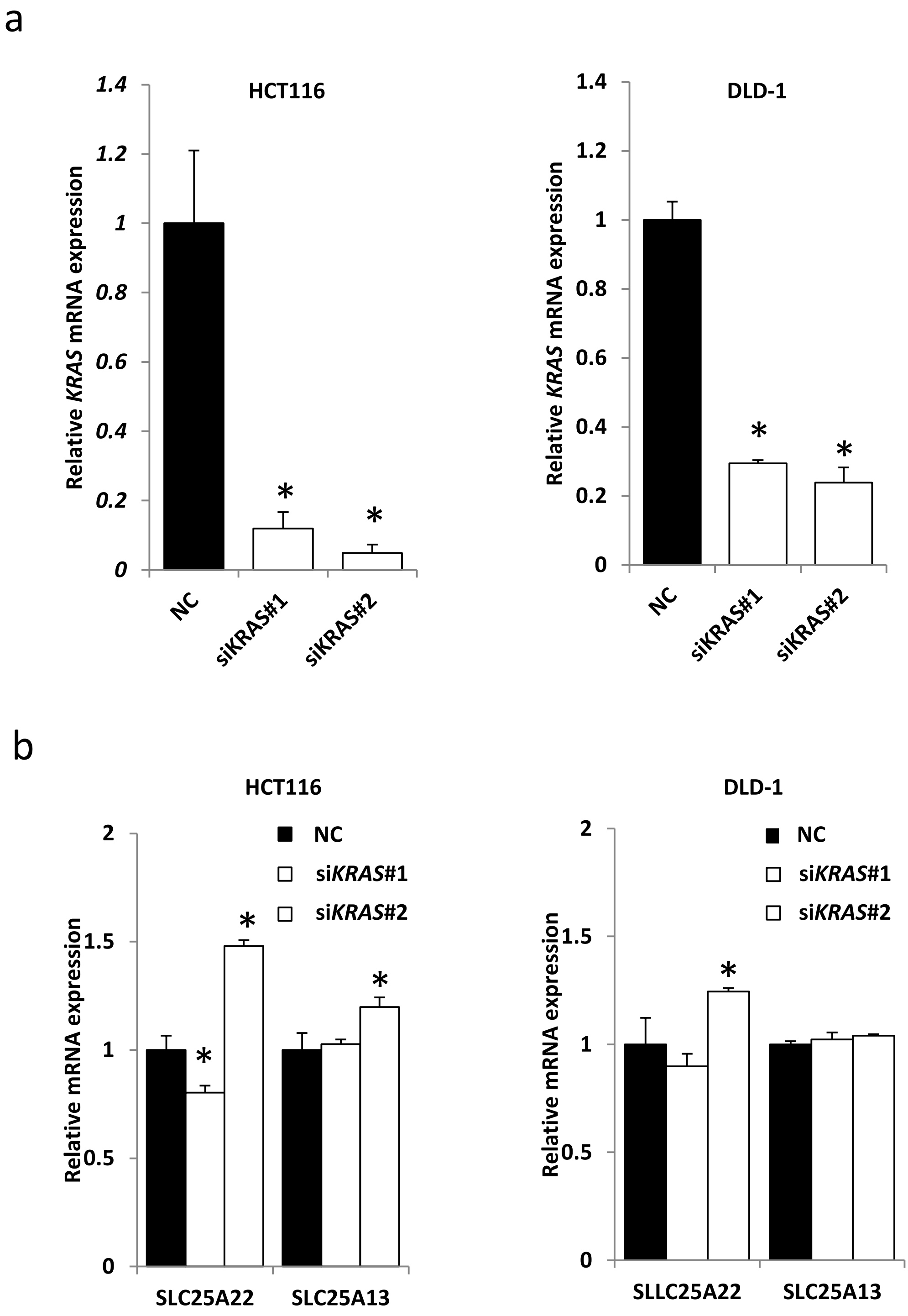

Figure S2. The effect of siSLC1A5 in colorectal cancer (CRC) cell lines. CRC cell lines were treated separately with two independent siRNA contructs (#1 and #2) targeting SLC1A5 and negative control (NC) siRNA and we performed western blotting 72hr after transfection.

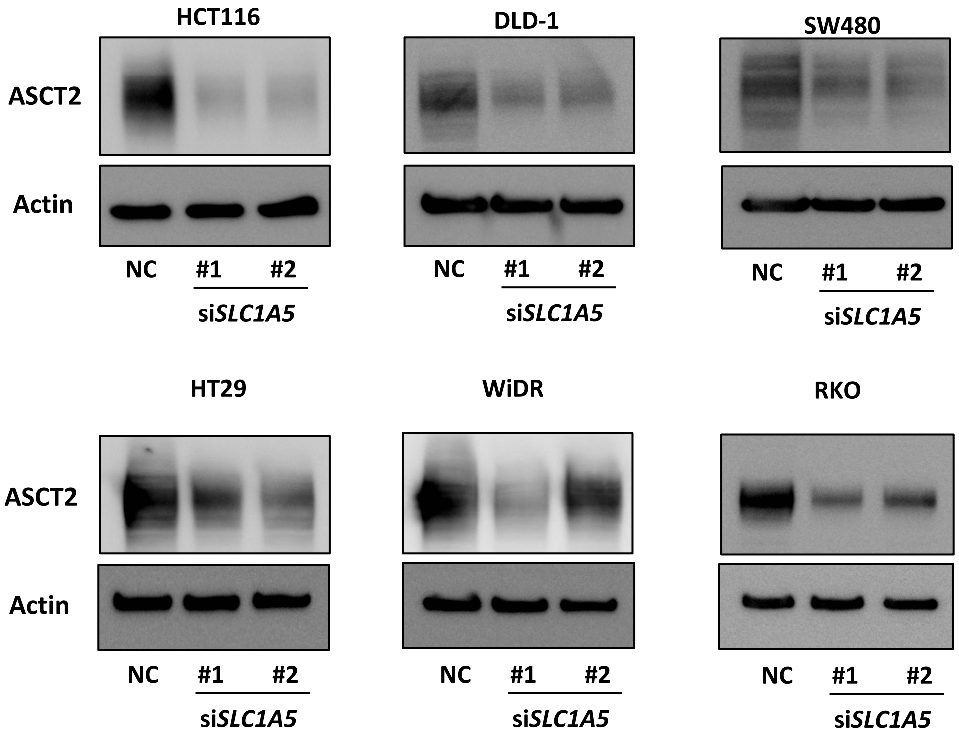

Figure S3. The effect of shSLC1A5 in HCT116. Quantitative RT-PCR (a) and western blotting (b) show the suppression of SLC1A5 (ASCT2) expression in HCT116.

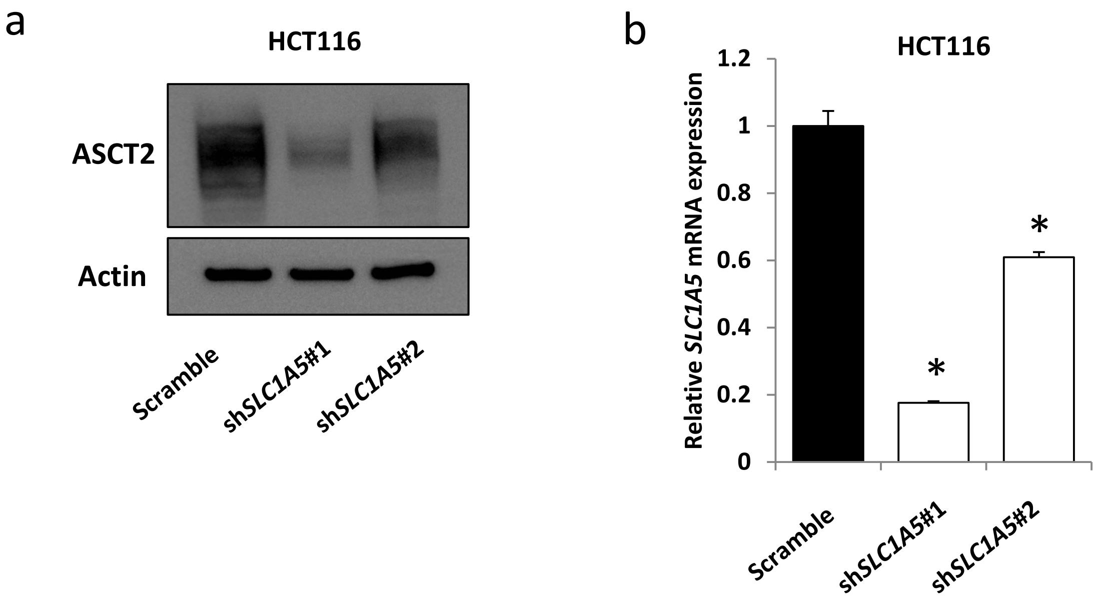

Supplement: Supplementary file 1 [file ijms-18-01632-s001.zip › ijms-210244-Supplementary figures.pdf]
